# Post Orgasmic Illness Syndrome (POIS) and Delayed Onset Muscle Soreness (DOMS): Do They Have Anything in Common?

**DOI:** 10.3390/cells10081867

**Published:** 2021-07-23

**Authors:** Balázs Sonkodi, Zsolt Kopa, Péter Nyirády

**Affiliations:** 1 Department of Health Sciences and Sport Medicine, University of Physical Education, 1123 Budapest, Hungary; 2 Department of Urology, Semmelweis University, 1082 Budapest, Hungary; kopa.zsolt@med.semmelweis-univ.hu (Z.K.); nyirady.peter@med.semmelweis-univ.hu (P.N.)

**Keywords:** post orgasmic illness syndrome, delayed onset muscle soreness, repeated bout effect, muscle spindle, Piezo 2 ion channel, acute compression proprioceptive axonopathy

## Abstract

Post orgasmic illness syndrome is a rare, mysterious condition with an unknown pathomechanism and uncertain treatment. The symptoms of post orgasmic illness syndrome last about 2–7 days after an ejaculation. The current hypothesis proposes that the primary injury in post orgasmic illness syndrome is an acute compression proprioceptive axonopathy in the muscle spindle, as is suspected in delayed onset muscle soreness. The terminal arbor degeneration-like lesion of delayed onset muscle soreness is theorized to be an acute stress response energy-depleted dysfunctional mitochondria-induced impairment of Piezo2 channels and glutamate vesicular release. The recurring symptoms of post orgasmic illness syndrome after each ejaculation are suggested to be analogous to the repeated bout effect of delayed onset muscle soreness. However, there are differences in the pathomechanism, mostly attributed to the extent of secondary tissue damage and to the extent of spermidine depletion. The spermidine depletion-induced differences are as follows: modulation of the acute stress response, flu-like symptoms, opioid-like withdrawal and enhanced deregulation of the autonomic nervous system. The longitudinal dimension of delayed onset muscle soreness, in the form of post orgasmic illness syndrome and the repeated bout effect, have cognitive and memory consequences, since the primary injury is learning and memory-related.

## 1. Introduction

Post orgasmic illness syndrome (POIS) is a rare, mysterious, but most likely under-reported, debilitating chronic condition with unknown pathomechanism and uncertain treatment. The name of POIS was introduced and described first by Waldinger and Schweitzer [1]. It was considered by Waldinger as an autoimmune reaction induced by specific cytokines to prostatic tissue produced seminal fluid [2]; however the study of Jiang et al. [3] could not confirm this hypothesis. The symptoms are flu-like and evolve immediately or hours after ejaculation with extreme fatigue, generalized weakness, foggy head, painful heavy leg and arm muscles, concentration and attention difficulties and irritation [2,4]. POIS last about 2–7 days after an ejaculation [2,4]. POIS presents a variable clinical picture in the intensity, durations, type of symptoms and their order of appearance [5,6]. Several pathophysiological hypotheses have been proposed to explain the condition, such as immunological phenomenon [5,7,8,9], opioid-like withdrawal [3], neuroendocrine response [10], transient deregulation of the autonomic nervous system [11], hypersensitivity and disordered cytokines [4,6]. 

This manuscript proposes to the scientific and medical community to consider an analogous primary injury mechanism in POIS, suggested by the new acute compression proprioceptive axonopathy theory of delayed onset muscle soreness (DOMS) [12], as one possible pathomechanism.

The cause of DOMS has been a mystery for more than 100 years [12]. The symptoms are delayed onset soreness, muscle stiffness, swelling, loss of force-generating capacity, reduced joint range of motion and decreased proprioceptive function [13]. The pain of DOMS is not felt for about 8 h, culminates in 1 or 2 days [14], and subsides within 7 days [15] after an unaccustomed or strenuous eccentric exercise activity [12]. Several pathophysiological hypotheses have been proposed to explain DOMS, like lactic acid, muscle spasm, connective tissue damage, muscle damage, inflammation and enzyme efflux theories [16]. 

However, “the general consensus amongst researchers is that one theory cannot explain the onset of DOMS. As a result, some researchers have proposed unique sequences of events in order to explain the DOMS phenomenon” [16]. Correspondingly, we propose that no single theory could explain POIS either. Interesting is to note the correlation between the hypotheses of DOMS and POIS; for example, transient deregulation of the autonomic nervous system, immunological phenomenon, disordered cytokines and neuroendocrine responses seem to be common characteristics of both conditions. 

Accordingly, the current hypothesis is not intended to challenge any current theories. It is, rather, about the critical path in the pathomechanism, which critical mechanism comes first and a reconsideration, in hindsight, of current ideas. Looking at the pathomechanism from a neurological angle may provide us with a better understanding, as such a mechanism is involved in the case of the acute compression proprioceptive axonopathy theory of DOMS [12]. Muscles and other tissues are only neuromodulators in this interpretation. The Piezo2 channels are the principal mechanotransducers of proprioception [17] and the acute mechanical stress-derived neuronal energy depleted impairment of Piezo2 channels at the proprioceptive sensory terminals are proposed to be the loci of microdamage in both DOMS and POIS. Furthermore, the acute depletion of spermidine is suggested to be an additional critical mechanism in the pathophysiology of POIS. Another interesting correlation is that many clinicians observed a lifelong premature ejaculation condition among their POIS patients [3,5,6,11] and foresee Piezo2 channels being implicated in the pathomechanism of premature ejaculation [18].

## 2. Repetitive Eccentric Contractions and Acute Compression Proprioceptive Axonopathy

According to a new theory, cognitive demand-derived unaccustomed or strenuous, eccentric or forced, lengthening contractions under an acute stress response (ASR) could cause microdamage to the proprioceptive sensory terminals of the muscle spindle in DOMS [12]. However, concentric contractions do not cause DOMS [19]. Notable is that Weerakkody et al. [20] demonstrated the contribution of large fiber sensory afferents to DOMS in 2001 [20,21]. Repetitive eccentric contractions are known to damage proprioception [22] and it is theorized that they could cause microdamage to the proprioceptive axon terminals in the muscle spindle [23]. This terminal microdamage is suggested to cause terminal arbor degeneration (TAD)-like mechano-energetic lesions on the peripheral ends of the pseudounipolar proprioceptive fibers [12]. 

Noteworthy is that this type of lesion, as experienced in paclitaxel-based chemotherapy, and proposed in DOMS, evolves in a dose-dependent, cumulative manner and is not associated with Wallerian-like axonal degeneration [24]. Accordingly, it has been suggested that paclitaxel has its microdamaging effect at the same sensory axon terminal locus [24], among others, where the mechano-energetic lesions of DOMS are proposed to evolve. This acute proprioceptive axonopathy is suggested to change the static encoding of the mostly unaffected stretch reflex of preprogrammed postural control by altering to a secondary compensatory pathway on the spinal dorsal horn [23]. Activated N-methyl-D-aspartate receptors (NMDAR) on the spinal dorsal horn are suggested to be the gate controllers of this second-messenger pathway on the central terminals of these pseudounipolar proprioceptive sensory neurons [23]. The compensatory effect could be exaggerated contractions due to dendritic persistent inward sodium current (NaPIC) inducement on motoneurons [23,25]. The NaPICs are suggested to be evoked due to the loss of static monosynaptic contributions from the microdamaged Type Ia sensory fibers [23]. This exchange of static monosynaptic connections to polysynaptic connections is proposed to be only represented in the delayed latency of the medium latency response (MLR) of the affected stretch reflex [23].

Repetitive eccentric contractions, as in DOMS-inducing exercise, are also executed during the erection process and the associated movements prior to ejaculation. Accordingly, the current hypothesis proposes the initiating role of an acute compression proprioceptive axonopathy of the muscle spindles in the pathomechanism of POIS. Premature ejaculation, an often-accompanied symptom of POIS [2], and other symptoms, could be indicative of this theory.

Erection and ejaculation are precisely orchestrated preprogrammed synchronized reflexes, that are induced by microcircuits and central pattern generators (CPG) on the spinal and supraspinal level that involve the autonomic nervous system and the neuroendocrine system. The detailed description of these mechanisms is not the subject of this paper, but highlighting the POIS-initiating critical neuronal microlesions that could disrupt and alter these precisely coordinated reflex activities. Current authors propose the important contribution of bulbospongiosus and ischiocavernosus muscles to these reflexes [26]. The bulbospongiosus muscle increases the erection of the glans, while the ischiocavernosus muscle enhances the lengthening flips [26]. Noteworthy is that erection is associated with elevated postural control of the penis, and lengthening flips entail eccentric contractions that are essential for DOMS development. Both of these muscles have small numbers of muscle spindles and their involvement in proprioception has been suspected [27]. Furthermore, the ventral insertion of the bulbospongiosus muscle, which is the superficial muscular layer of the perineum and pelvic floor, and the ischiocavernosus muscle, are unified both functionally and morphologically [28]. Both muscles contribute to seminal expulsion of ejaculation [29]. As in DOMS, it is hypothesized that an analog TAD like mechano-energetic proprioceptive terminal microlesion could prevail in the muscle spindles of these muscles during unaccustomed or strenuous eccentric contractions that comprise copulation or masturbation (see Table 1).

It is suggested that this acute compression proprioceptive axonopathy could affect the erection and ejaculation mechanism by diverting part of the monosynaptic static encoding of the stretch reflex to polysynaptic encoding, as is proposed in DOMS [23]. One consequence of this alteration could be dendritic PIC inducement on motoneurons that could result in exaggerated contractions [23]. Paradoxically, this heightened contracting activity could induce further compression on the bulbourethral glands and, as a result, the loss of monosynaptic pathways could possibly lead to premature ejaculation. These changes are induced on the spinal level in a way that is out of supraspinal or cognitive control in a repeated copulation or masturbation event executed within the 2–7 day timeframe. This repeated event is suggested to be the equivalent of the repeated bout effect (RBE) of DOMS, explained later in this paper. Noteworthy is that rhythmic clonic contractions of the bulbospongiosus muscles propelled by the ejaculation spinal pacemaker, a CPG, are the basis for the urethrogenital reflex [30,31]. The urethrogenital reflex is considered a cocontroller of the somatic and autonomic reflex expulsion of ejaculation [30]. 

In summary, repeated unaccustomed or strenuous forced lengthening contractions comprising copulation or masturbation activity within a week after the primary injury is proposed to cause possible premature ejaculation and POIS. The repeated event could lead to an impaired urethrogenital reflex; therefore, its delayed latency of the MLR could cause less control of the static stretch component. As a result, the involuntary somatic and autonomic component of the ejaculatory reflex is less opposed, and the expulsion is triggered by the distention of the urethra by the seminal fluids and by compression of the urethra by exaggerated contractions of the bulbospongiosus and ischiocavernosus muscles. Therefore, repeated microinjury of the proposed proprioceptive nerve terminals could lead to impairment of the intricate system that controls ejaculation. It is important to note that premature ejaculation certainly has other causes than the suggested microinjury mechanism, and not all POIS patients have premature ejaculation, probably due to individual variability in the number of mechano-sensors and NaPIC inducement. Furthermore, the contribution to the above phenomena from penile and skin proprioceptive neurons should not be excluded. There are proprioception receptors in the penis and deeper layers of the skin [32,33,34]. Skin and bone nerves are involved in, or theorized to contribute to, the stretch reflex in accordance with Hilton’s law [12,25,35]. Therefore, superposition of compression forces of eccentric contractions could also cause potential microdamage to these dermal somatosensory nerves with the same consequences, as is hypothesized in the muscle spindle of the bulbospongiosus and ischiocavernosus muscles. It has long been realized by Proske and Gandevia [22] that damaging eccentric exercises are to blame for the impairment of proprioception. Notable is that impaired involvement in skin proprioception may point towards underlying atopic constitution as was found in a POIS patient [3,6]. 

## 3. Acute Stress Response, Polyamines and the Opioid System

Inducement of an ASR during fatiguing unaccustomed or strenuous eccentric contractions is a crucial element of the new theory of DOMS [12], as well as and the current hypothesis. It serves as a driver when exercise performance is fatiguing, but cognitive demand dictates the sustainment of heightened exercise performance regardless of fatigue [23]. ASR, a complete withdrawal of parasympathetic tone, is proposed to be invoked by osteocalcin [36] in DOMS [12], but an ASR in the ejaculation phase of POIS is likely to be enhanced by the release of oxytocin as well [37,38]. It is noteworthy that cardiac autonomic full recovery from an ASR in DOMS, measured by heart rate variability, takes 24–48 h and overlaps the peak of the ascending phase of hyperalgesia in DOMS [14,23]. This ASR inducement could be the basis of the transient deregulation of the autonomic nervous system observed in POIS [11]. The minimal symptom duration of POIS [4], namely 2 days, also overlaps with the cardiac autonomic full recovery of DOMS measured by heart rate variability [39] and with the peak of DOMS hyperalgesia [14,23]. 

Polyamines, like spermidine and spermine, are well known signalers of bidirectional glial-neuronal communication, especially in periods of stress such as trauma [40]. Therefore, spermidine and spermine release could be an ASR modulator in the ejaculation phase of POIS. Moreover, spermidine and spermine have several beneficial effects, such as increasing longevity, cell proliferation and differentiation, channel regulation, modulation of learning and memory, antinociception and neuroprotection, and also have antidepressant and antioxidant domains [40]. An ASR-related depletion of spermidine, elevated osteocalcin and oxytocin, and impaired cross-activation of primary afferents and sympathetic fibers in the muscle spindle could inform the hypothesis concerning transient deregulation of the autonomic nervous system [11]. Noteworthy is that muscle spindles can contain sympathetic fibers [12,41] and compression microinjury of the primary afferents in the muscle spindle could impair the cross-talking between these fibers. 

Spermidine and spermine also interact with the opioid system by facilitating or disrupting opioid-induced reward [42]. Spermidine depletion-induced opioid-like withdrawal could be an additional stress source that could augment the ASR during ejaculation and could explain the behavior and cognitive symptoms of POIS. Important to note is that in human and animal studies, oxytocin is antinociceptive on the mu- and kappa-opioid receptors in the brain [43,44]. In addition, the cross-modulation of the opioid and endocannabinoid system might play a role [45,46,47]. Noteworthy is that anandamide, an endocannabinoid neurotransmitter, has an effect on CB2 peripheral cannabinoid receptors and on the central nervous system, but anandamide has a functional uptake process in the prostate as well [48]. It is no surprise that opioid-like withdrawal has been hypothesized in the pathophysiology of POIS [3].

Overall, if the repetitive eccentric contractions prior to ASR inducement are unaccustomed or strenuous, then mechano-energetic TAD-like microdamage of the proprioceptive axon terminals could prevail as a result of an ASR during ejaculation Hence, the ASR is not only theorized to provide a window of opportunity for the mechano-energetic proprioceptive axon terminal lesion but, as a result, initiates the transient deregulation of the autonomic nervous system and could facilitate the depletion of spermidine that could disrupt the opioid-induced reward system. 

Putrescine, another polyamine, is an important component of the seminal plasma and has a role in seminal clot formation [29]. However, putrescine is an important precursor for spermidine and GABA synthesis in adrenal glands [49]. Therefore, the loss of putrescine with seminal plasma could be a strong local indirect spermidine/spermine depletion signal pathway. Polyamines, such as spermidine and spermine, have an important neuroprotective function, especially in neuronal stress and trauma [40]; therefore, this strong signal pathway could result in a temporary polyamine homeostatic imbalance under an ASR [40]. The first critical impact of this imbalance could be on NMDAR regulation. Acute compression axonopathy is hypothesized to activate NMDARs, but polyamines are supposed to have a role in the rectification of NMDA regulation [23,40]. Therefore, the excessive demand and depletion of spermidine could lead to further homeostatic imbalance in the central nervous system. It is hypothesized that spermidine demand resulting from loss of putrescine would provide insufficient signaling, but the proposed concomitant neuronal microtrauma could be a strong signaling pathway in POIS that cannot be buffered by the available spermidine in the central nervous system. 

It is noteworthy that symptoms do not evolve in POIS patients during sexual activities in the absence of ejaculation [4]. In accordance with the current POIS and DOMS hypothesis, the ASR seems to be essential in order to develop acute compression proprioceptive axonopathy and resultant spermidine depletion resulting in POIS. It is important that the presence of an ASR is not preprogrammed in the ejaculation process, as opposed to the preprogrammed increase of sympathetic activity. Therefore, the presence of an ASR, proposed to be induced by unaccustomed or strenuous eccentric contractions during the ejaculatory process, is a window of opportunity for the suggested neuronal microdamage, but not the preprogrammed heightened sympathetic activity.

## 4. Activated NMDARs and Low Grade Neuroinflammation 

The time-frame of POIS symptoms [2] may overlap not only the time frame of DOMS symptoms, but the 7-day time frame of increased delayed blood-spinal cord barrier (BSCB) and blood-brain barrier (BBB) permeability in a peripheral nerve injury (PNI) [2,12,50]. Furthermore, there is evidence of transient and permanent neuropathy of the pudendal nerve if it is compressed or stretched [51]. It has been demonstrated that peripheral nerve injury is irreversible if the pudendal nerve is stretched by more than 12% [51]. The branches of the pudendal nerve innervate the bulbospongiosus and ischiocavernosus muscles. In the case of POIS, only an acute temporary terminal proprioceptive axonopathy is proposed as the muscle spindle related locus of compression microinjury due to repetitive unaccustomed or strenuous forced lengthening contractions. 

Spermidine is an anti-inflammatory metabolite, but the depletion of it is suggested to facilitate a delayed low-grade peripheral neuroinflammation process caused by the proposed acute compression sensory terminal axonopathy in the muscle spindles. BSCB and BBB permeability start to increase six hours after the onset of the terminal microlesion [12,50], and this is when the delayed onset of pain evolves in DOMS due to activated NMDARs on the spinal dorsal horn [14,15,23]. An analogous mechanism is suggested in POIS, causing its delayed onset of symptoms [4]. The depletion of spermidine also seems to play a critical role in the bidirectional glial-neuronal communication in the central nervous system and in the onset of low-grade neuroinflammation. Furthermore, the activated NMDARs in a spermidine depleted state could cause symptoms of POIS, including irritability and a foggy feeling in the head [1].

Viruses and host cells compete for polyamines because polyamines are crucial for both [52] and, as a result, virus infections deplete spermidine in the host cells with flu-like symptom inducement. A current theory suggests that spermidine release enhances olfactory memory and learning [53] during the preprogrammed ejaculation phase of copulation or masturbation, and flu-like symptom are proposed to arise due to ASR-induced spermidine depletion. 

## 5. The Repeated Bout Effect

DOMS has a longitudinal dimension, namely the repeated bout effect (RBE). If the same or similar eccentric or forced lengthening exercise bout is repeated, it is remembered for up to a year [54]. It is suggested of the RBE mechanism that certain memory pathways are involved, such as working memory, episodic memory, inflammation memory, immune memory and pain memory [23]. POIS could have an equivalent longitudinal dimension involving initial acute compression proprioceptive axonopathy (see Table 1), but in POIS the same exercise bout is nearly always repeated within one year of the relevant memory time range. Stress-induced changes in bidirectional glial-neuronal communication could cause glial cells to contribute to learning and memory [23,55]. Spermidine and spermine, like osteocalcin and oxytocin, not only could have a role in ASR modulation, but could be the central players in learning and opening the suggested memory pathways [42,56]. Spermidine has a facilitatory role on acquisition and early consolidation of memory, but not on retrieval and late consolidation [57]. Noteworthy is that late consolidation of memory starts six hours after training [57], which is when the BSCB and BBB permeability start to increase in a PNI [50] and when the delayed onset of pain evolves in DOMS [14,15,23]. 

## 6. Ontogenetic Relevance

The ontogenetic importance of muscle spindles has been emphasized by their playing an essential role in growth [12]. The grounds of this theory were laid down by Hilton’s Law [12]. The proprioceptive sensory terminals of the muscle spindles seem to have a more central role than is currently viewed. Profound life-sustaining, genetically preprogrammed hardwiring could be affected by their proposed mechano-energetic microlesions, which could have relevance in DOMS, noncontact injuries, neurodegeneration, oncology, autoimmune diseases, pain management and sexual dysfunction [12,23]. Not letting these proprioceptive nerve terminals regenerate or return to their preprogrammed functioning, as well as elongated loading of the secondary compensatory pathway, could have progressing consequences. 

## 7. Testing This Hypothesis and Possible Interventions

Up to a year of abstinence from copulation or masturbation among POIS patients would be an interesting test for the hypothesis. However, it is very likely that the involvement of the opioid system and, later, the endocannabinoid system in the signaling mechanism of POIS have longer memory time ranges than the RBE. Therefore, this treatment option might be successful only if POIS is diagnosed very early after the primary injury. Dietary spermidine supplementation could be tested as an option prior to sexual activities. The first successful treatment has been reported with human chorionic gonadotropin [58], which seems to substantiate spermidine depletion in the etiology [59]. Moreover, Reisman presented successful treatment in 57% of POIS patients with the highly selective alpha 1 A-blocker, Sidosin [1]. The findings of Reisman could be explained by Sidosin improving blood flow and the respiratory capacity of the affected proprioceptive nerves, preventing the neuroenergetic TAD-like lesion at the terminals of these nerves in a high percentage of patients. The preventive use of Riluzole, an approved life-lengthening drug for amyotrophic lateral sclerosis treatment, prior to sexual activity could be another possibility based on its action mechanism [23,60]. 

## 8. Cellular Mechanism of the Critical Primary Injury

The cellular locus of the above-mentioned potential primary microinjury has been highlighted by the term “watch this space” in relation to muscle spindles and the possible role of proprioceptive mechanotransduction Piezo2 channels triggered by forced lengthening contractions [61]. Piezo proteins are enormous channels with 120 to 160 transmembrane segments [62]. However, their detailed topology, pore formation, mechanical force detection and gating mechanism are barely known [62].

We hypothesize that repetitive unaccustomed or strenuous, forced lengthening contractions and an ASR could induce energy depletion at the hyperexcited primary afferent’s peripheral terminal, and the resulting dysfunctional mitochondria could impair glutamate vesicular release and proprioceptive mechanotransduction Piezo2 ion channels. This theoretical mechanism could explain the TAD-like lesion of the primary afferents and the glutamate excitotoxicity of DOMS as the primary injury [12,23]. 

It is noteworthy that the role of Piezo2 channels has been demonstrated in premature ejaculation rats, with the involvement of inward currents [18], although we suggest that the mechanism is related to peripheral nerve sensitization and not to TAD-like lesions. Accordingly, premature ejaculation related to mechanical hypersensitivity involves significantly increased Piezo2 in the penis head and in the dorsal root ganglion (DRG) [18] without mechano-energetic lesions. However, in the case of POIS, and underlying DOMS, it is theorized that the Piezo2 channels of the proprioceptive axon terminals of the muscle spindle in the bulbospongiosus and ischiocavernosus muscles suffer an acute stress induced mechano-energetic lesion. As a result, the static encoding of the bulbourethral reflex could be impaired, leading to premature ejaculation in POIS. Noteworthy is that Piezo2 mechanotransducers have been identified as the main proprioceptor channels in mouse models [17], further supporting the current POIS and DOMS hypotheses. Moreover, the possible role of microinjured Piezo2 channels in sensory neurons regulating the distension of the urethra should not be ruled out [63].

The proprioceptive primary afferent sensory neurons in the muscle spindle are pseudounipolar cells. Their cell bodies are located in the DRG, and their axons are split into two branches. The peripheral branch is terminated in the muscle spindle, while their central branch is terminated on the spinal dorsal horn. The peripheral ending is hypothesized to go through a TAD-like lesion in DOMS [12]. Bennett et al. described TAD as, “if the energy deficiency is severe enough then degeneration happens, and the threshold for degeneration will be lowest in the neuronal compartment that has the highest energy requirement” [24]. Furthermore, they suggested that the sensory axonal ending is the compartment with the highest energy requirement [24]. Accordingly, the superposition of compression forces by lengthening contractions under an ASR could possibly cause such a severe mechano-energetic insult on axonal mitochondria in the primary sensory neurons of the muscle spindles impairing the axon’s energy supply [12]. The terminal arbor of the primary sensory afferents in the muscle spindle, packed with mitochondria [64], have been implicated as an analog neuronal compartment that could undergo TAD-like lesion leading to DOMS [12]. 

Noteworthy is that DOMS has a dichotomous injury mechanism where the primary damage is theorized to be ASR-induced acute compression proprioceptive axonopathy, and the secondary injury is harsher tissue damage due to the loss of proprioception [12,65,66]. The tissue damage, including the muscle damage, is conveyed by Type IV nerve fibers or C-fibers [19]. The involvement of C-fibers in the mechanical hyperalgesia of DOMS is only secondary to the microinjured proprioceptive fibers [67]. Nevertheless, only C-fibers could contribute to slow temporal summation [67]. It is likely that there is no, or limited, secondary tissue microinjury in POIS because the repetitive eccentric contractions are often terminated right after the ejaculatory moment. Therefore, the amount of unaccustomed and strenuous, eccentric contractions under an ASR is limited, if any, which means the temporal summation of mechanical hyperalgesia cannot evolve full-blown due to the lack, or limited contribution, of C-fibers (see Table 1). Noteworthy is that Weerakkody et al. [21] presumed that these two kinds of nociceptive pathways are processed differently by the central nervous system, but the large fiber-related pain contribution to DOMS is the one subject to a central switch. Moreover, the involvement of Piezo2 to mechanical hyperalgesia has been also implicated [62] and is consistent with the primary proprioceptive terminal microdamage theory of the current hypothesis and with the new acute compression proprioceptive axonopathy theory of DOMS. Furthermore, it was demonstrated that the magnitude of the mechanical hyperalgesia of DOMS is not limited to the microinjured muscle area and does not reflect on the magnitude of muscle damage [19]. Another important phenomenon is that the delayed onset of pain is typically felt during muscle stretch and contraction, but not at rest, during the approximately one week long DOMS [67]. Therefore, the lengthening flips by ischiocavernosus muscles during the erection process could be slightly painful within 2–7 days after primary neuronal microinjury. However, the pain sensation could be minimal if secondary muscle damage is limited after the ejaculation process. Accordingly, the symptoms of POIS entail muscle tension in the back or neck, muscle weakness, painful muscles, heavy legs and stiffness in muscles, similar to the experience with DOMS [1,12,23] without full blown mechanical hyperalgesia. It is important to highlight that the involved symptomatic muscles contribute to postural control, and the involvement of these muscular symptoms is proposed to be due to impaired proprioception. 

POIS was considered by Waldinger as an auto-immune reaction induced by specific cytokines to seminal fluid produced by prostatic tissue [2]; however, the study of Jiang et al. [3] could not confirm this hypothesis. We suggest that this proposed “auto-immune reaction” is the result of acute compression proprioceptive axonopathy-induced neuroinflammation, which is equivalent to the secondary injury phase of DOMS when harsher tissue damage prevails. Accordingly, when parasympathetic activity returns fully after an ASR, immune-mediated inflammation comes into play in the microinjured tissues with the involvement of cytokines, but is part of a normal regenerative mechanism in order to restore homeostasis [12]. In summary, we theorize that acute stress-induced acute mitochondrial energy depletion steers the way to acute Piezo2 channelopathy that eventually evolves into a TAD-like lesion or acute compression proprioceptive axonopathy. As a result, the central terminals of the pseudounipolar sensory afferents also go through pathological changes. Activated NMDARs are proposed to be the gate controllers of these changes [23]. Bewick et al. suggested that glutamate was released from synaptic-like vesicles in the mechano-sensory terminals of the muscle spindles in a stretch-modulated manner [68], but Than et al. demonstrated that glutamate release is, indeed, vesicular [61,69]. We propose that acute stress-induced energy deficiency could impair glutamate vesicular release leading to glutamate spillover at the peripheral terminal arbor. Moreover, the dysfunctional Piezo2 channels could become leaky even to glutamate. Eventually, glutamate excitotoxicity could activate NMDARs at presynaptic central endings at the spinal dorsal horn [23]. 

## 9. Conclusions

According to our hypothesis, the primary injury of POIS could be an acute compression proprioceptive axonopathy in the muscle spindles of the bulbospongiosus and ischiocavernosus muscles, as is suspected in delayed onset muscle soreness (DOMS). The TAD-like lesions of DOMS are theorized to result from acute stress-related, energy-depleted, dysfunctional mitochondria-induced impairment of Piezo2 ion channels and glutamate vesicular release. The recurring symptoms of POIS after each ejaculation is suggested to be analogous to the RBE of DOMS. However, there are differences in the pathomechanism, mostly attributed to the extent of secondary tissue damage and to the extent of spermidine depletion. Individual differences in the genetic encoding of the Piezo2 ion channels should not be ruled out.

## Figures and Tables

**Table 1:** The theoretical injury phases of POIS and DOMS.

Acute Piezo2 Channelopathy	Acute Compression Proprioceptive Axonopathy	
**Post orgasmic illness syndrome** symptoms 2 days	**Post orgasmic illness syndrome** symptoms 3–7 days	**Delayed onset muscle soreness** symptoms 7 days
**PRIMARY INJURY PHASE**		
Repetitive unaccustomed or strenuous eccentric contractions		
Fatigue-induced acute stress response		
Ejaculation	Ejaculation	
Energy depletion of the mitochondria in the primary afferent terminal of the muscle spindle		
Impairment of glutamate vesicular release and Piezo2 channels		
Spermidine depletion	Spermidine depletion	
**SECONDARY INJURY PHASE**		
No tissue damage	Limited tissue damage	Harsher tissue damage
No C-fiber contribution	C-fiber contribution limited	C-fiber contribution
**TERTIARY INJURY PHASE**		
Post orgasmic illness syndrome = Repeated bout effect	Post orgasmic illness syndrome = Repeated bout effect	Repeated bout effect
